# S73. EFFECT OF LURASIDONE ON COGNITION IN ADOLESCENTS WITH SCHIZOPHRENIA: INTERIM ANALYSIS OF A 2-YEAR OPEN-LABEL EXTENSION STUDY

**DOI:** 10.1093/schbul/sby018.860

**Published:** 2018-04-01

**Authors:** Philip D Harvey, Robert Goldman, Michael Tocco, Ling Deng, Josephine Cucchiaro, Antony Loebel

**Affiliations:** 1 University of Miami Miller School of Medicine; 2 Sunovion Pharmaceuticals, Inc

## Abstract

**Background:**

The onset of schizophrenia typically occurs during adolescence or early adulthood and is characterized by severe symptomatology and lifelong functional impairment. Cognitive dysfunction is common in schizophrenia and is associated with significant impairment in functioning. Very little is known about the long-term effects of antipsychotic treatment on cognition in adolescents with schizophrenia. To this extent, cognitive data are presented from an interim analysis of an ongoing 2-year long-term safety study of lurasidone in the treatment of children and adolescents with schizophrenia.

**Methods:**

Patients aged 13–17 years with schizophrenia who completed 6 weeks of double-blind (DB), placebo-controlled treatment with lurasidone were enrolled in a 2-year, open-label (OL) study in which patients were continued on lurasidone or switched from placebo to lurasidone. Cognitive function was assessed with the Brief CogState battery, which evaluates four cognitive domains: processing speed, attention/vigilance, visual learning, and working memory. Based on normative data, an overall cognitive composite Z-score was calculated as the average of the standardized Z-scores for each of the four cognitive domains. These results are based on an interim analysis of the 2-year data.

**Results:**

A total of 271 patients completed 6 weeks of double-blind treatment and entered the 2-year extension study. At the time of the interim analysis, 132 patients had completed 52 weeks of treatment (24 patients were 2-year study completers; 96 patients were still ongoing; and 12 patients had discontinued after 52 weeks); 57 patients were still ongoing in the first 1-year of treatment; and 82 patients terminated prior to week 52. The cognitive composite Z-score showed impairment at double-blind baseline (-1.09). Mean change in Z-score, from DB baseline to OL weeks 0 (OL-baseline), 28, 52, and 104, respectively, were observed for the cognitive composite (+0.04, +0.16, +0.30, +0.57), and for the CogState domains processing speed (-0.08, +0.02, +0.16, +0.68), attention/vigilance (0.00, 0.00, +0.05, +0.38), visual learning (+0.19, +0.45, +0.75, +1.07), working memory accuracy (+0.18, +0.24, +0.73, +0.30), working memory speed (+0.06, +0.23, +0.28, +0.15).

**Discussion:**

In this study of adolescents with schizophrenia, lurasidone was not associated with cognitive impairment after up to 104 weeks of treatment. Larger sample sizes are needed to confirm the robustness of the improvement was observed in selected cognitive domain scores, most notably visual learning and processing speed.

